# Analysis of enteric nervous system and intestinal epithelial barrier to predict complications in Hirschsprung’s disease

**DOI:** 10.1038/s41598-020-78340-z

**Published:** 2020-12-10

**Authors:** Anne Dariel, Lucie Grynberg, Marie Auger, Chloé Lefèvre, Tony Durand, Philippe Aubert, Catherine Le Berre-Scoul, Aurélien Venara, Etienne Suply, Marc-David Leclair, Philine de Vries, Guillaume Levard, Benoit Parmentier, Guillaume Podevin, Françoise Schmitt, Véronique Couvrat, Sabine Irtan, Erik Hervieux, Thierry Villemagne, Hubert Lardy, Carmen Capito, Cécile Muller, Sabine Sarnacki, Jean-François Mosnier, Louise Galmiche, Pascal Derkinderen, Hélène Boudin, Charlène Brochard, Michel Neunlist

**Affiliations:** 1 University of Nantes, INSERM, TENS, The Enteric Nervous System in Gut and Brain Diseases, IMAD, Nantes, France; 2 Paediatric Surgery Department, La Timone-Enfants Hospital, Assistance Publique des Hôpitaux de Marseille, 264 rue Saint Pierre, 13385 Marseille, France; 3 Paediatric Surgery Department, University Hospital of Nantes, Nantes, France; 4 Paediatric Surgery Department, University Hospital of Brest, Brest, France; 5 Paediatric Surgery Department, University Hospital of Poitiers, Poitiers, France; 6 Paediatric Surgery Department, University Hospital of Angers, Angers, France; 7 Paediatric Surgery Department, Hospital of Le Mans, Le Mans, France; 8 Paediatric Surgery Department, Armand Trousseau Hospital, Paris, France; 9 Paediatric Surgery Department, University Hospital of Tours, Tours, France; 10 Paediatric Surgery Department, Necker Enfants Malades Hospital, Paris, France; 11 Pathology Department, University Hospital of Nantes, Nantes, France; 12 Pathology Department, Necker Enfants Malades Hospital, Paris, France

**Keywords:** Enteric neuropathies, Enteric nervous system, Biomarkers, Paediatric research, Translational research, Biomarkers, Predictive markers

## Abstract

In Hirschsprung’s disease (HSCR), postoperative course remains unpredictable. Our aim was to define predictive factors of the main postoperative complications: obstructive symptoms (OS) and Hirschsprung-associated enterocolitis (HAEC). In this prospective multicentre cohort study, samples of resected bowel were collected at time of surgery in 18 neonates with short-segment HSCR in tertiary care hospitals. OS and HAEC were noted during postoperative follow-up. We assessed the enteric nervous system and the intestinal epithelial barrier (IEB) in ganglionic segments by combining immunohistochemical, proteomic and transcriptomic approaches, with functional ex vivo analysis of motility and para/transcellular permeability. Ten HSCR patients presented postoperative complications (median follow-up 23.5 months): 6 OS, 4 HAEC (2 with OS), 2 diarrhoea (without OS/HAEC). Immunohistochemical analysis showed a significant 41% and 60% decrease in median number of nNOS-IR myenteric neurons per ganglion in HSCR with OS as compared to HSCR with HAEC/diarrhoea (without OS) and HSCR without complications (*p* = 0.0095; *p* = 0.002, respectively). Paracellular and transcellular permeability was significantly increased in HSCR with HAEC as compared to HSCR with OS/diarrhoea without HAEC (*p* = 0.016; *p* = 0.009) and HSCR without complications (*p* = 0.029; *p* = 0.017). This pilot study supports the hypothesis that modulating neuronal phenotype and enhancing IEB permeability may treat or prevent postoperative complications in HSCR.

## Introduction

Hirschsprung’s disease (HSCR) is a rare disease (1 in 5000 live births) and a life-threatening developmental disorder that results from an incomplete colonisation of the embryonic gut by enteric neuronal and/or glial precursors affecting mainly the distal gut^1^. The absence of enteric ganglia over a variable length of distal intestine induces a tonic contraction of the involved segment leading to functional obstruction, usually in the first days of life^2^. The current treatment is the surgical removal of the aganglionic bowel followed by anastomosis to the ganglionic zone considered ‘healthy’. Despite successful surgery, postoperative Hirschsprung-associated enterocolitis (HAEC) occurs in 25% of patients and is the first cause of mortality^3^. Furthermore, functional obstructive symptoms (OS) and/or intestinal transit disorders occur in up to 45% of patients in their long-term outcomes^4,5^. HSCR-associated postoperative evolution is currently unpredictable, mainly because the physiopathological mechanisms underlying the gastrointestinal motility disorders still remain unknown. Therefore, a better characterisation of these mechanisms could result in the identification of predictive markers of postoperative complications and also ultimately the identification of novel therapeutic targets.

We therefore undertook a prospective multicentre study to define predictive factors of the postoperative complications OS and HAEC in HSCR patients by evaluating the enteric nervous system (ENS) and the intestinal epithelial barrier (IEB) in the ‘healthy’ ganglionic segment at the time of curative surgery, combining immunohistochemical, biochemical and transcriptomic approaches, together with functional ex vivo analysis.

## Results

### Patient characteristics and prospective follow-up

Characteristics of the 18 HSCR patients and the 16 patients with anorectal malformations (ARM) were comparable: age at surgery (*p* = 0.12) (52 days [6;222] and 128 days [0;284], respectively), gestational age at birth (*p* = 0.15) (38 weeks [36;39] and 38 weeks [35;39], respectively), birth weight (3352 g [2160;4250] and 3045 g [2475;3700], respectively), and gender (*p* = 1) (16 males and 15 males, respectively). There was no associated malformation in the HSCR patients. ARM patients were considered in our study as a comparison group. Ten of the 18 HSCR patients (55.6%) presented postoperative complications unrelated to surgery and detailed in Table 1 (median follow-up: 23.5 months [17;39]): 6 OS, 4 HAEC (2 with OS) and 2 with diarrhoea (D) (without HAEC or OS). OS were diagnosed at a median age of 21 months in patients without associated HAEC and 3 months in patients with HAEC. A transanal pull-through was performed in all 18 HSCR patients (Swenson-like n = 5; Soave-like n = 13). Complications were comparable whatever the type of transanal pull-through: 3 complications with Swenson-like pull-through (3 OS, 1 HAEC) and 7 with Soave-like pull-through (3 OS, 3 HAEC) (*p* = 1). No ARM patients developed enterocolitis.

**Table 1:** Postoperative complications in HSCR patients and age of onset: obstructive symptoms (OS), Hirschsprung-associated enterocolitis (HAEC) and diarrhoea.

Patient	Stool consistency	Number of stools	OS				HAEC				Diarrhoea
			Yes/No	Age at onset of OS (months)	Type	Treatment	Yes/No	Age at HAEC (months)	HAEC score	HAEC severity	Yes/No
1	C	4/day	Yes	26	Faecal loading	Laxatives	No	–	–	–	No
2	C	2–3/day	Yes	21	Abdominal distension, faecal loading	–	No	–	–	–	No
3	D	2/week	Yes	19	Straining for defecation, constipation	Laxatives	No	–	–	–	No
4	D	2/week	Yes	16	Straining for defecation, constipation	Diet	No	–	–	–	No
5	A and B	1–4/day	Yes	2	Abdominal distension, faecal loading	Washouts, botox, laxatives	Yes	21	10	2	No
6	B	2/day	Yes	4	Abdominal distension, faecal loading	Washouts, micro-enemas	Yes	4	11	2	No
7	B	1–2/day	No	–	–	–	Yes	2.5 and 4	7 and 9	2 and 2	No
8	C	1–2/day	No	–	–	–	Yes	8.5	7	1	No
9	A	8–10/day	No	–	–	–	No	–	–	–	Yes
10	A	5–7/day	No	–	–	–	No	–	–	–	Yes

The treatment received in case of OS was noted regardless of its efficiency. Stool consistency was evaluated with the Amsterdam stool form scale for not-toilet-trained infants^6^ (A = watery, B = soft, C = formed, D = hard). Episodes of HAEC were diagnosed according to the HAEC score (HAEC score ≥ 4)^7,8^ and severity was graded from 1 to 3 according to published guidelines^3^.

### Predictive factor of obstructive symptoms

#### Morphological characteristics and neurochemical phenotype of the MP

As myenteric neurons are key regulators of GI motility, we first aimed to determine whether changes in the myenteric plexus (MP) morphology or neurochemical coding (at the time of curative surgery) could be used as predictive factors of postoperative motility dysfunctions in HSCR, and particularly for those with OS. Therefore, these parameters were compared between three subgroups of HSCR patients: HSCR with OS (n = 6), HSCR with HAEC or D (without OS) (n = 4), and HSCR without complications (n = 8). Furthermore, we also compared in this study the three HSCR subgroups with an additional group of ARM patients (n = 16), a comparison group that we were able to constitute at the neonatal and early childhood period. ARM is a congenital colorectal malformation with distinct postoperative complications, mainly bowel continence disorders related to abnormal anal sphincter and pelvic muscular complex inherent to this developmental disorder, but probably unrelated to an underlying GI motility or ENS dysfunction as observed in HSCR. Thus, we considered that ARM could be used to determine specific biomarkers of HSCR postoperative complications.

**Figure 1:**
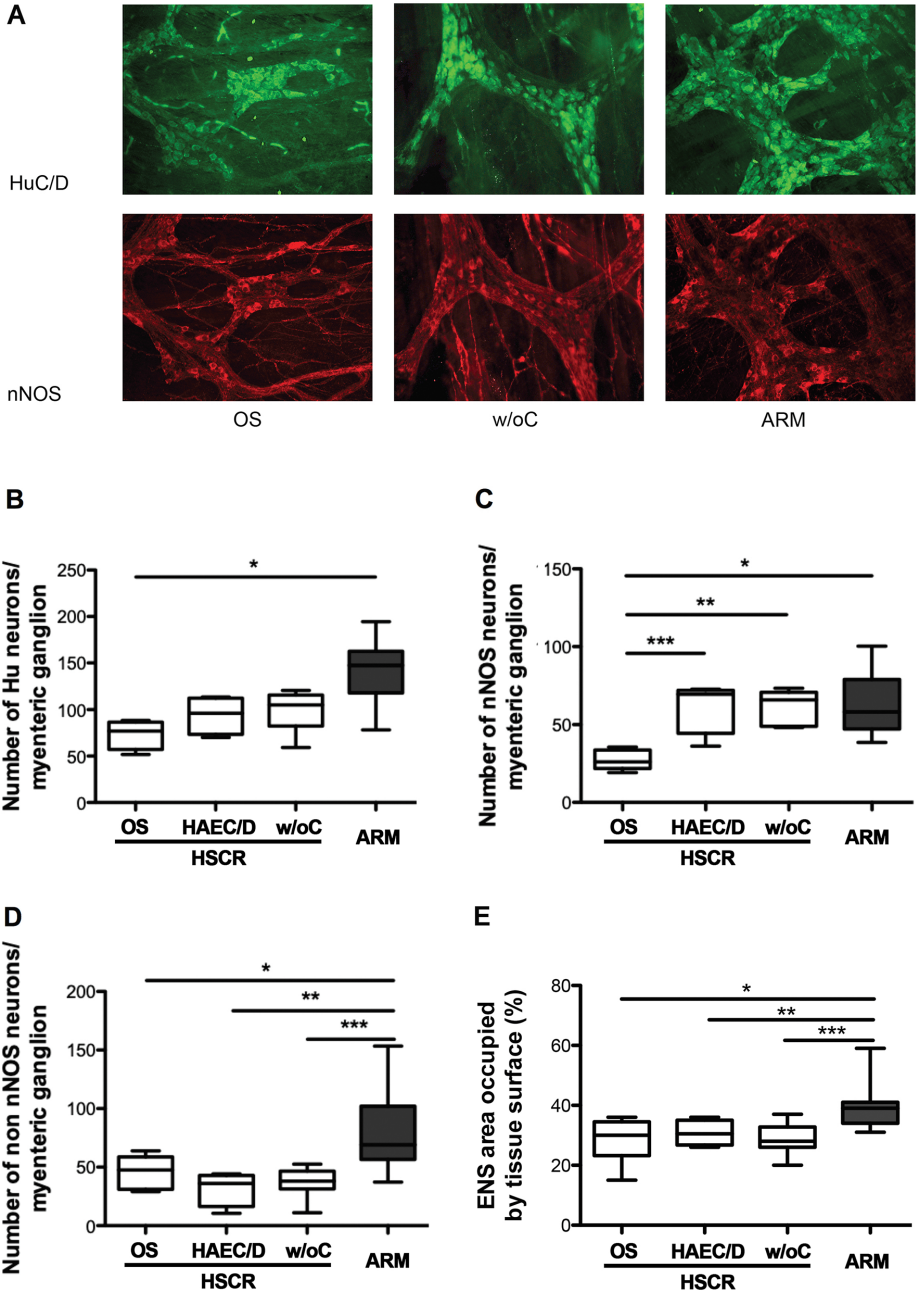
Immunohistochemical analysis of myenteric plexus in the 3 HSCR subgroups (white bars) (HSCR patients with obstructive symptoms (OS), with HAEC or diarrhoea (HAEC/D) and without complications (w/oC)) and in ARM (grey bar) patients. (**A**) Immunohistochemical labeling of myenteric plexus with anti-HuC/D and anti-nNOS antibodies (Scale bar = 100 μm). (**B**) Number of HuC/D-IR myenteric neurons per ganglion (*p = 0.0011). (**C**) Number of nNOS-IR myenteric neurons per ganglion (*p = 0.0095; **p = 0.002; ***p = 0.006). (**D**) Number of non nNOS-IR myenteric neurons per ganglion (*p = 0.019; **p = 0.009; ***p = 0.002). (**E**) ENS area occupied by tissue surface (*p = 0.007; **p = 0.02; ***p = 0.001). Medians with ranges. ENS: enteric nervous system.

First, we aimed to determine whether changes in neuronal density and neurochemical coding might predict postoperative OS complications. The number of HuC/D-immunoreactive (IR) myenteric neurons per ganglion was not different between the three HSCR subgroups (*p* = 0.09) (Fig. 1A,B). However, when ARM was compared to the three HSCR subgroups, the number of HuC/D-IR myenteric neurons per ganglion was significantly different (*p* = 0.0026). In particular, quantitative analysis showed a significant 48% decrease in the number of HuC/D-IR myenteric neurons per ganglion in HSCR with OS as compared to ARM (*p* = 0.0011).

Next, we analysed the neurochemical phenotype of myenteric neurons by studying two specific subpopulations: neuronal nitric oxide synthase (nNOS)-IR neurons and calretinin-IR neurons. Quantitative analysis showed significant 41% and 60% decreases in the number of nNOS-IR myenteric neurons per ganglion in HSCR with OS, as compared to HSCR with HAEC/D, and HSCR without complications (*p* = 0.0095 and *p* = 0.002, respectively) (Fig. 1A–C). Interestingly, the number of nNOS-IR myenteric neurons per ganglion was comparable between HSCR with HAEC/D, HSCR without complications and ARM (*p* = 0.95). We also expressed changes of the nitrergic subpopulation as changes in the proportion of nNOS over the total number of myenteric neurons (identified with HuC/D; % (nNOS  /Hu +) myenteric neurons). Using this representation, we showed a significant increase in the proportion of nNOS +/Hu + myenteric neurons in HSCR patients with HAEC/D (63% [52;87]) and in HSCR patients without complications (61% [55;81]) as compared to ARM patients (51% [21;60]) (*p* = 0.02 and *p* = 0.02, respectively). However, the proportion of nNOS myenteric neurons was decreased in HSCR patients with OS (41% [25;46]) as compared to HSCR patients with HAEC/D and without complications (*p* = 0.0095 and *p* = 0.007, respectively).

In contrast, the number of calretinin-IR neurons per myenteric ganglion was comparable between the three HSCR subgroups (*p* = 0.98) and also with ARM (*p* = 0.93). As choline acetyltransferase (ChAT)-IR in our preparation was too faint to be evaluated rigorously, we measured expression levels of acetylcholine by Elisa in tissue of longitudinal muscle/myenteric plexus (LMMP). We did not find any difference between the three HSCR subgroups (*p* = 0.82) and with ARM (*p* = 0.9). Finally, the number of non-nNOS-IR myenteric neurons per ganglion was significantly different between ARM and each of the three HSCR subgroups (*p* = 0.0015) but it was similar between the three HSCR subgroups (*p* = 0.45) (Fig. 1D).

Besides the changes in the ENS phenotype, we also analysed morphological parameters on whole-mount preparations of LMMP. Although the percentage of tissue area occupied by ENS structures (defined by analysing Calret-IR ganglionic and interganglionic structures) was comparable between the three HSCR subgroups (*p* = 0.87), it was statistically smaller in each of the three HSCR groups as compared to ARM (Fig. 1E). Furthermore, although the neuronal cytoplasmic area was comparable between the three HSCR subgroups (*p* = 0.26), it was larger in each of the three HSCR subgroups (HSCR with OS, HSCR with HAEC/D, HSCR without complications) as compared to ARM (*p* = 0.013, *p* = 0.006 and *p* = 0.006, respectively) (data not shown).

#### Ex vivo measurements of neuromuscular transmission

**Figure 2:**
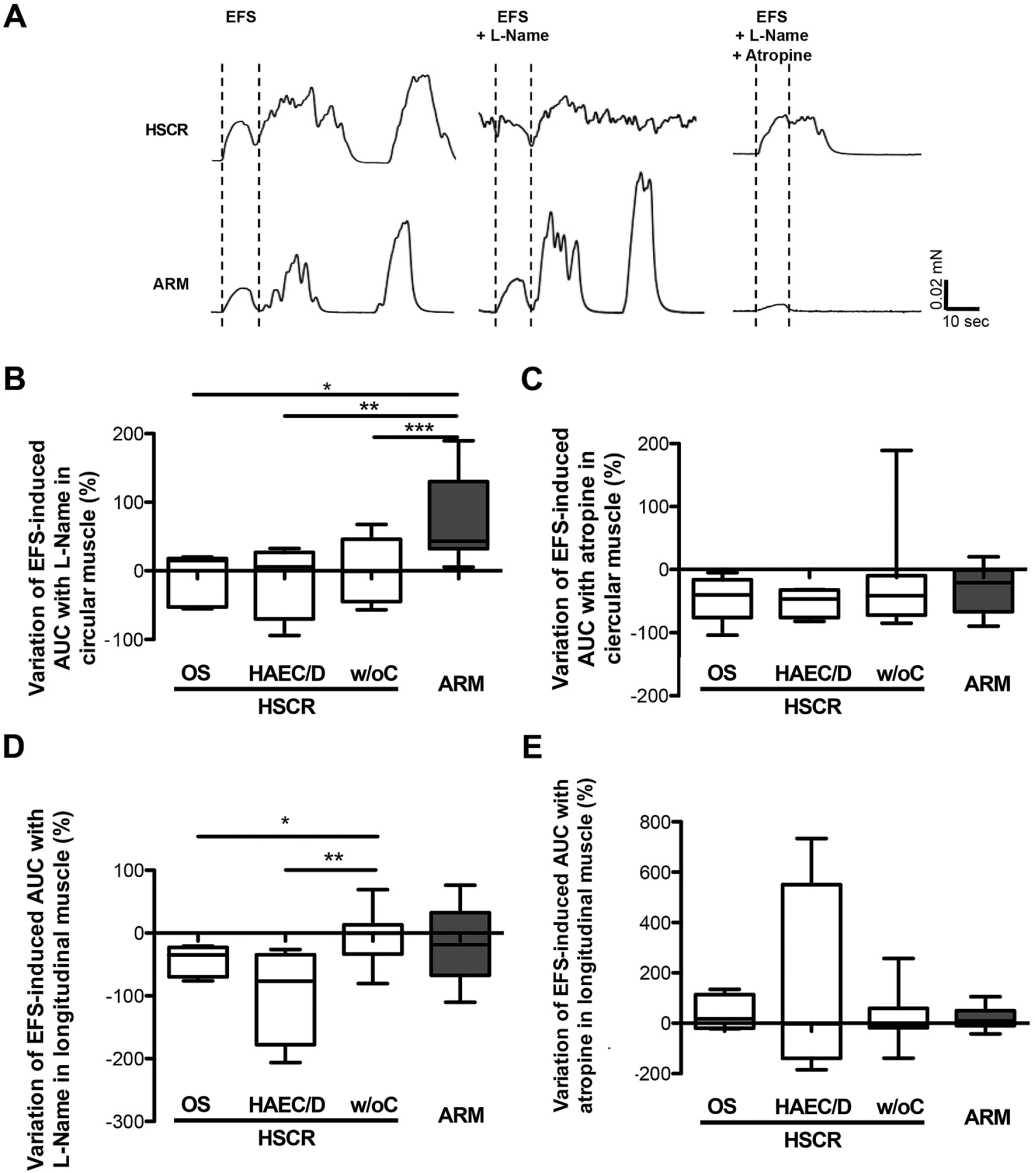
Neuromuscular transmission was assessed in colonic circular muscle strips in the 3 HSCR subgroups (white bars) (HSCR patients with obstructive symptoms (OS), with HAEC or diarrhoea (HAEC/D) and without complications (w/oC)) and in ARM (grey bar) patients. (**A**) EFS-contractile response in basal condition, with l-Name, with l-Name + atropine. (**B**) Variation of EFS-induced AUC with l-Name (*p = 0.006; **p = 0.02; ***p = 0.02), and (**C**) with l-Name + atropine. (**D**) Variation of basal tension level with l-Name, and E) with l-Name + atropine (*p = 0.007; **p = 0.014; ***p = 0.02). Medians with ranges. *EFS* electrical field stimulation, *AUC* area under the curve.

We then investigated whether changes observed in the ENS phenotype, in particular in the population of nNOS-IR neurons, could impact neuromuscular contractile responses in the four previously defined groups. Colonic circular and longitudinal muscle strips were stimulated by electrical field stimulation (EFS) and the EFS-induced contractile response was analysed in basal condition, after addition of N-nitro-l-arginine methyl ester (l-Name) and after addition of atropine (Fig. 2A).

First, we assessed that, in basal condition, the EFS-induced area under the curve (AUC) in circular and longitudinal muscles were comparable between the 3 HSCR subgroups (*p* = 0.46 and *p* = 0.78, respectively) and the ARM group (*p* = 0.69 and *p* = 0.76, respectively).

Next, in circular muscle, we showed that the variation of EFS-induced AUC with l-Name was comparable between the three HSCR subgroups (*p* = 0.94), but was smaller in each of the three HSCR groups compared to ARM (*p* = 0.05) (Fig. 2B). In particular, this variation of EFS-induced AUC with l-Name was + 43.2% in ARM and tended to or was significantly higher compared to HSCR with OS (+ 14.8%) (*p* = 0.006), HSCR with HAEC/D (+ 5.5%) (*p* = 0.02), and HSCR without complications (− 0.8%) (*p* = 0.02). In contrast, the variation of EFS-induced AUC with atropine was comparable between the three HSCR groups (*p* = 0.81) and also with ARM (*p* = 0.67) (Fig. 2C).

Finally, in longitudinal muscle, we showed that the variation of EFS-induced AUC with l-Name was statistically different between the three HSCR subgroups (*p* = 0.026), and tended to be different with the ARM group (*p* = 0.065) (Fig. 2D). In particular, this variation of EFS-induced AUC with l-Name was − 0.1% in HSCR without complications and was significantly higher compared to HSCR with OS (-34.9%) (*p* = 0.049), and HSCR with HAEC/D (− 76.6%) (*p* = 0.028). In contrast, the variation of EFS-induced AUC with atropine was comparable between the three HSCR groups (*p* = 0.83) and also with ARM (*p* = 0.88) (Fig. 2E).

#### Expression levels of glial fibrillary acidic protein (GFAP)

**Figure 3:**
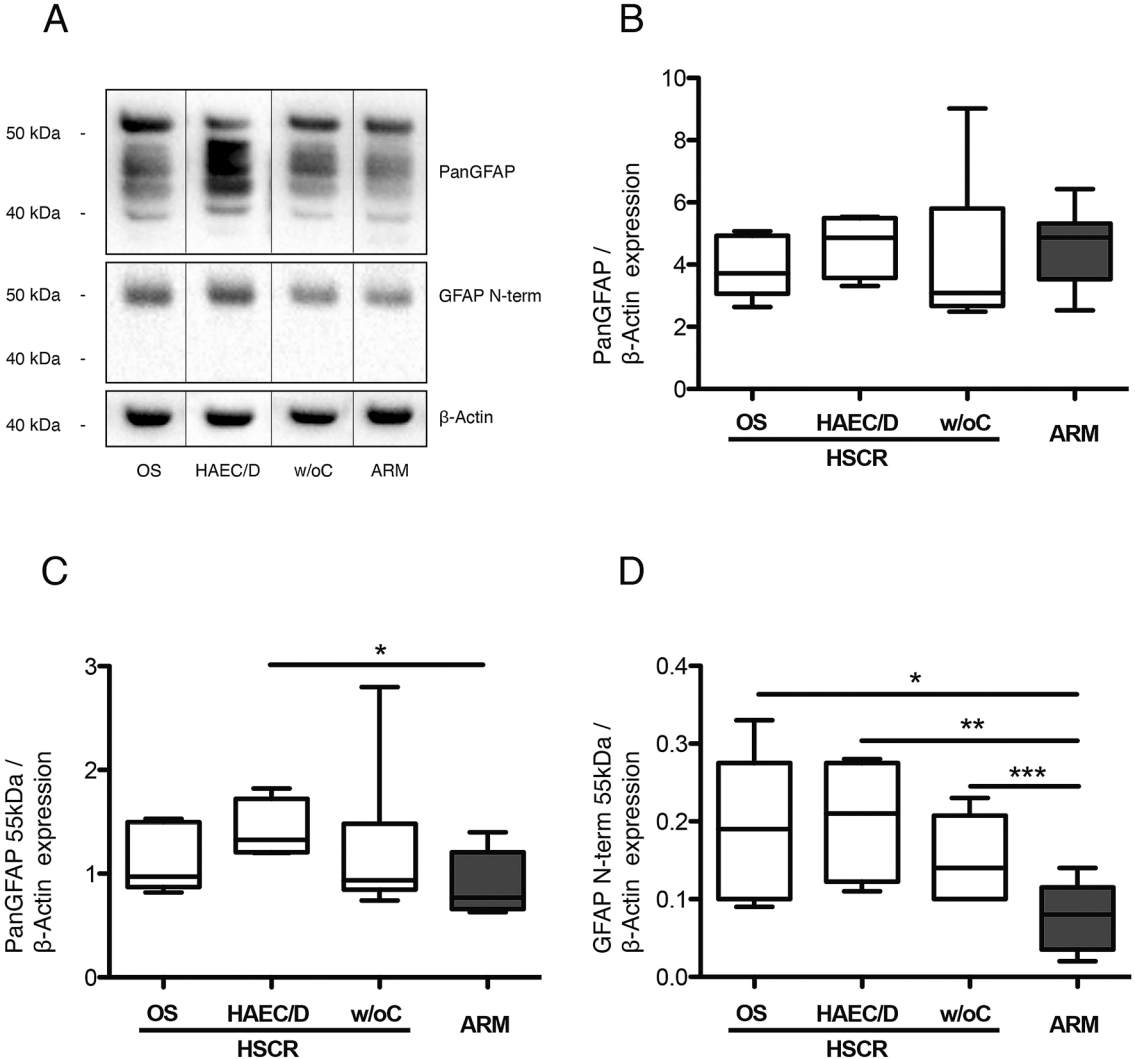
Glial fibrillary acidic protein (GFAP) expression in the muscular layer of sigmoid colon in the 3 HSCR subgroups (white bars) (HSCR patients with obstructive symptoms (OS), with HAEC or diarrhoea (HAEC/D) and without complications (w/oC)) and in ARM (grey bar) patients. The dividing lines in (**A**) delineate the position of the cropping performed on the same gel before grouping.. Full-length blots are in Supplementary Material. (**A**) Immunoblot analysis using an antibody recognizing all GFAP isoforms and trunked forms of the protein (panGFAP) and one specific antibody recognizing intact GFAP isoforms (GFAP N-term). All samples derive from the same blot. (**B**) PanGFAP expression (all bands and 55 kDa band), (**C**) PanGFAP 55 kDa band expression (*p = 0.02), and (**D**) GFAP N-term expression by Western Blot analysis (*p = 0.027; **p = 0.01; ***p = 0.015). Medians with ranges.

As enteric glial cells (EGC) have recently been suggested to play a central role in motility dysfunctions, we next aimed at determining the putative existence of glioplastic changes in the LMMP that might predict postoperative OS complications in HSCR patients. We evaluated the expression levels of GFAP by Western Blot with the PanGFAP antibody that recognises a large majority of the known isoforms and truncated forms of the protein. One prominent band migrating at 55 kDa and five faster migrating bands (between 40 and 50 kDa) were detected (Fig. 3A). Density analysis of all six GFAP-IR bands did not show any difference between the four groups (Fig. 3B). However, density analysis of the 55-kDa band showed a higher expression in HSCR with OS, and HSCR with HAEC/D, than in ARM (*p* = 0.08 and *p* = 0.02, respectively) (Fig. 3C), but no difference was detected between the three HSCR subgroups.

The 55-kDa GFAP band is likely to represent full-length GFAP, while the lower-molecular-weight bands are N-terminal truncated forms of the protein^9^. To confirm further that the 55-kDa GFAP band represents full-length GFAP, we used the GFAP N-term antibody, which recognises intact GFAP but not its amino-terminal cleavage products. This antibody detected the full-length 55-kDa GFAP in HSCR and ARM patients (Fig. 3A). Quantification showed that the immunoreactivity of this 55-kDa band was significantly higher in each of the three HSCR subgroups compared to ARM, but no differences were detected between the three HSCR subgroups (*p* = 0.54) (Fig. 3D).

#### Expression level of tool-like receptors (TLRs)

Recent evidence has shown that TLRs, in particular TLR2 and TLR4, are expressed on the ENS and can regulate ENS maturation and development^10^. The mRNA expression of TLR2 and TLR4 was comparable between HSCR with OS, HSCR with HAEC/D, and HSCR without complications (*p* = 0.42 and *p* = 0.35, respectively). However, the expression of TLR2 and TLR4 was significantly reduced in the HSCR group with OS compared to ARM (*p* = 0.026 and *p* = 0.018, respectively) (data not shown). In addition, we found significant positive correlations between the expression of TLR2 and the number of nNOS-IR neurons per ganglion (r = 0.44; 95% IC 0.04–0.71; *p* = 0.03), and also the expression of TLR4 and the number of nNOS neurons per ganglion (r = 0.45; 95% IC 0.06–0.72; *p* = 0.02) (data not shown).

### Predictive factor of Hirschsprung-associated enterocolitis

#### Ex vivo paracellular and transcellular permeabilities

Paracellular and transcellular permeabilities were compared between the three HSCR subgroups and ARM: HSCR patients with HAEC (n = 4), HSCR patients with OS or D (without HAEC) (n = 6), HSCR patients without complications (n = 8), and ARM (n = 16).

**Figure 4:**
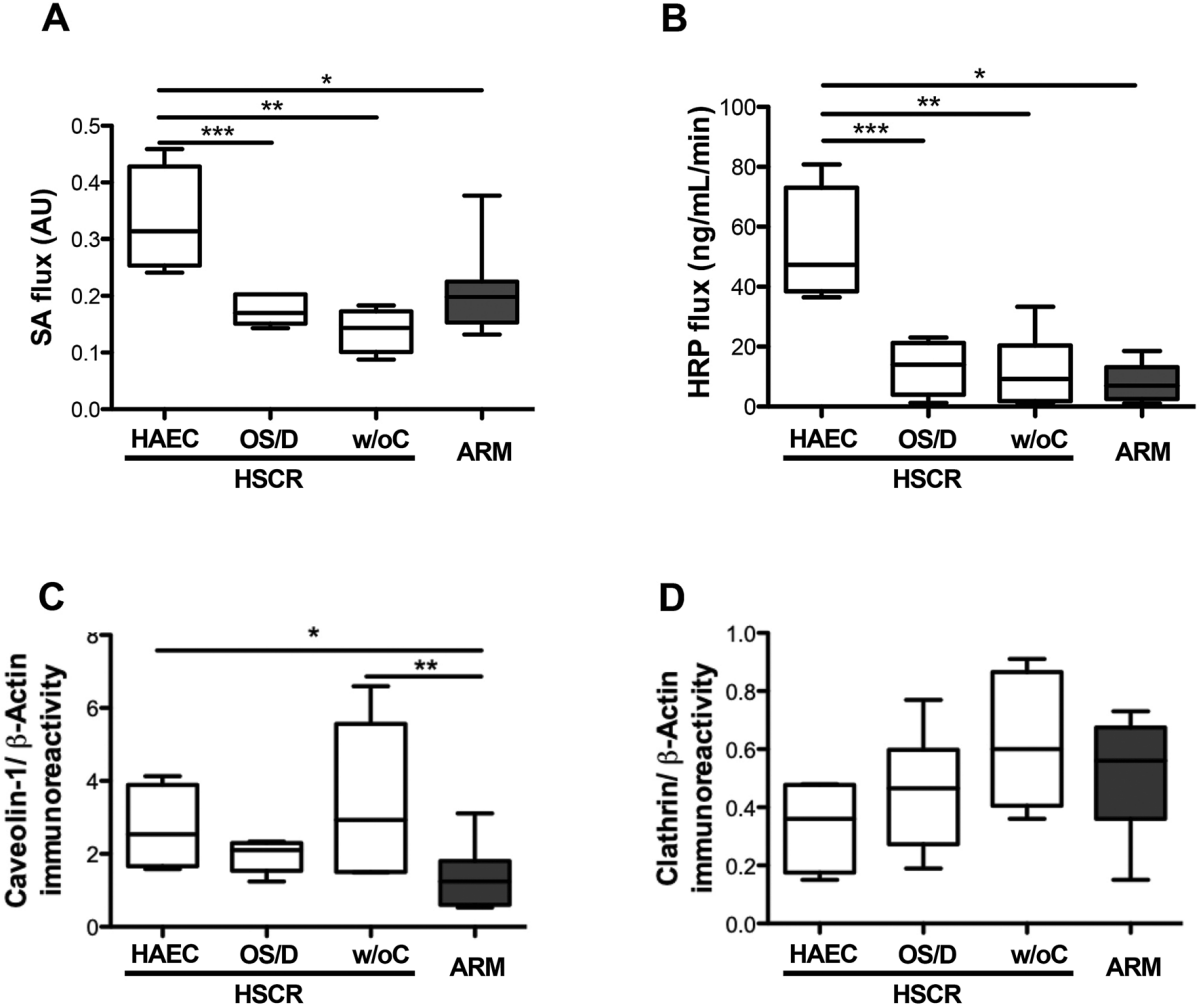
Paracellular and transcellular permeabilities in the 3 HSCR subgroups (white bars) (HSCR patients with Hirschsprung-associated enterocolitis (HAEC), with obstructive symptoms or diarrhoea (OS/D) and without complications (w/oC)) and in ARM (grey bar) patients. (**A**) Paracellular permeability measured with sulfonic acid (SA) flux (*p = 0.02; **p = 0.0095; ***p = 0.016), and (**B**) transcellular permeability measured with and Horse Radish Peroxydase (HRP) flux (*p = 0.013; **p = 0.017; ***p = 0.029). Mucosal expression of (**C**) caveolin-1 (*p = 0.03; **p = 0.04) and (**D**) clathrin in Western Blot. Medians with ranges. AU: arbitrary unit.

Interestingly, paracellular permeability analysis showed significant 84% (*p* = 0.016) and 118% (*p* = 0.0095) increases in sulfonic acid (SA) flux in HSCR with HAEC as compared to HSCR with OS/D, and HSCR without complications, respectively (Fig. 4A). In addition, transcellular permeability analysis also showed significant 238% (*p* = 0.029) and 415% (*p* = 0.017) increases in the horseradish peroxidase (HRP) flux in HSCR with HAEC compared to HSCR with OS/D, and HSCR without complications, respectively (Fig. 4B).

Next, we analysed the mucosal expression levels of key tight–junction (TJ) proteins involved in the control of paracellular permeability by Western Blot. No significant changes in the expression of ZO-1 (*p* = 0.95), occludin (*p* = 0.81), JAMA (*p* = 0.84), cingulin (*p* = 0.75), or claudin-1 (*p* = 0.57) were observed between the three HSCR subgroups. The expression of caveolin-1 and clathrin, proteins involved in the control of transcellular permeability, were also comparable between the three HSCR subgroups (*p* = 0.27, *p* = 0.63, respectively). However, caveolin-1 expression was significantly higher, or tended to be higher, in each of the three HSCR subgroups as compared to ARM (Fig. 4C,D).

#### Mucosal expression of inflammatory cytokines and TLR

**Figure 5:**
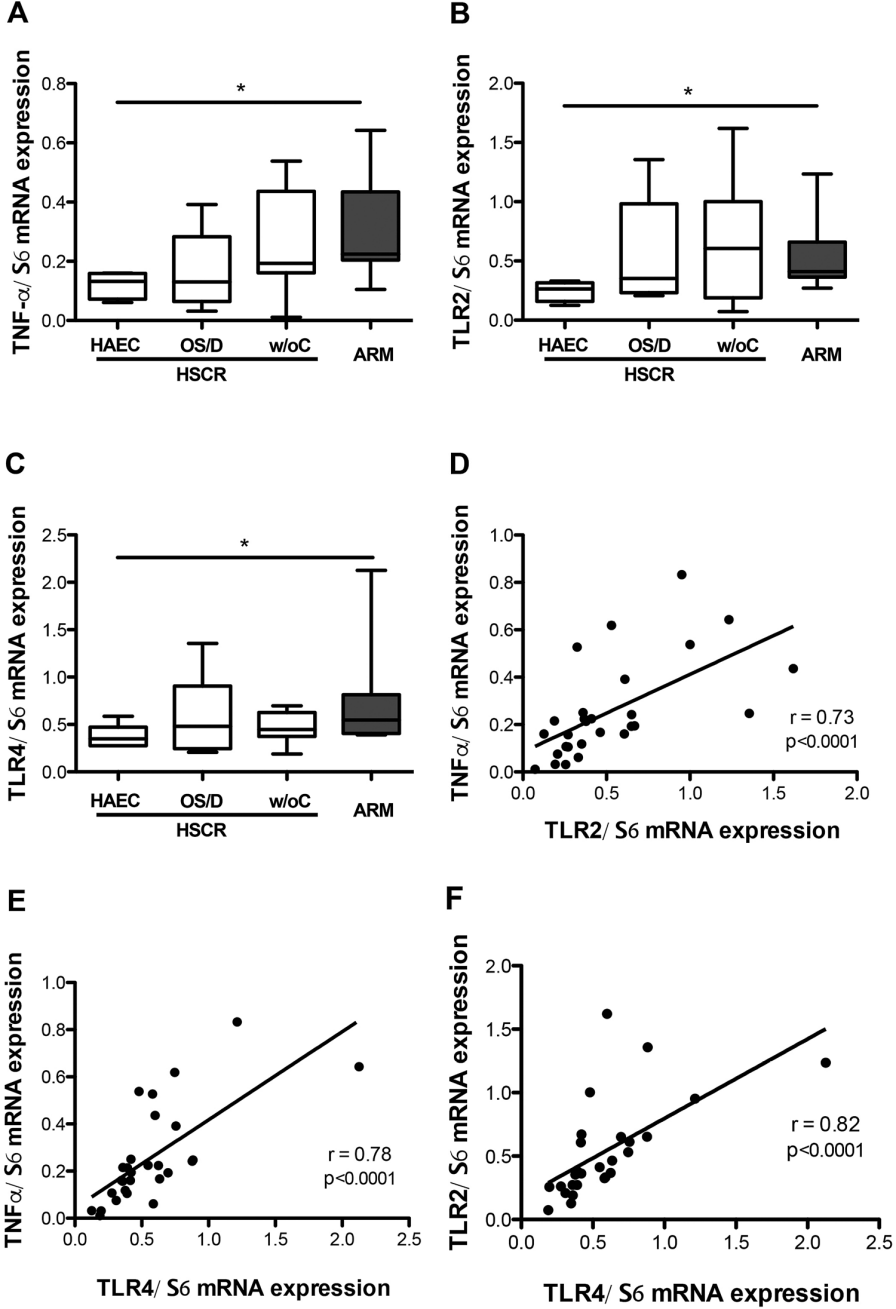
Expression of (**A**) TNFα expression (*p = 0.02), (**B**) TLR2 expression (*p = 0.01), and (**C**) TLR4 expression (*p = 0.02) in qPCR in the 3 HSCR subgroups (white bars) (HSCR patients with Hirschsprung-associated enterocolitis (HAEC), with obstructive symptoms or diarrhoea (OS/D) and without complications (w/oC)) and in ARM (grey bar) patients. Medians with ranges. (**D**) Correlation between TNFα and TLR2 expressions (r = 0.73; 95% IC 0.48–0.87; *p* < 0.0001), (**E**) between TNFα and TLR4 expressions (r = 0.78; 95% IC 0.56–0.89; *p* < 0.0001), and (**F**) between TLR4 and TLR2 expressions (r = 0.82; 95% IC 0.63–0.92; *p* < 0.0001).

In a final part of this study, we aimed to determine whether mucosal mRNA expression of inflammatory cytokines could be predictive of HAEC postoperative complications in HSCR. No significant changes were observed in the expression of inflammatory cytokines IL1β (*p* = 0.76), IL8 (*p* = 0.30), IL10 (*p* = 0.76), IFNγ (*p* = 0.16) or TNFα (*p* = 0.22) between the three HSCR subgroups. The expression of TLR2, TLR4, TLR9 were also comparable between the three HSCR subgroups (*p* = 0.40, *p* = 0.43, *p* = 0.83, respectively) (Fig. 5A–C). Nevertheless, the expression of TNFα (*p* = 0.02), TLR-2 (*p* = 0.01) and TLR-4 (*p* = 0.02) were significantly decreased in the HSCR group with HAEC compared to ARM. A positive correlation was noted between the expression of TLR2 and TNFα, TLR4 and TNFα and finally TLR2 and TLR4 (Fig. 5D–F).

## Discussion

In this prospective multicentre study, we hypothesised that the analysis of the ENS phenotype and gut functions in the normoganglionic segment following surgery for HSCR could be a source of biological or functional markers that could be distinctively associated with postoperative complications. We identified neuroplastic changes in the ENS characterised by a reduced number of NOS-IR neurons per myenteric ganglion specifically in HSCR patients with postoperative OS as compared to HSCR patients devoid of postoperative OS. In addition, we also identified a significant increase in paracellular and transcellular permeability in HSCR patients who presented postoperative HAEC in comparison with HSCR without HAEC. Such factors, once validated in larger cohorts, could be used not only to better stratify patients at risk of complications in order to propose them an oriented postoperative follow-up, but also to develop novel therapeutic approaches aimed at reducing risks of developing these complications, in particular by restoring ENS or gut functions.

The first major novel findings are that we identified in the normoganglionic segments of HSCR patients (at the time of curative surgery) distinct functional and biological markers that were associated with the occurrence of the two major postoperative complications: OS and HAEC.

Concerning postoperative OS, we revealed that the number of nNOS-IR neurons per myenteric ganglion was significantly reduced in the normoganglionic area of HSCR patients who presented postoperative OS as compared to other HSCR subgroups without OS (HSCR patients with HAEC/diarrhoea, or without complications). In addition, in HSCR with OS, the number of nNOS neurons was significantly reduced as compared to ARM. However, the number of nNOS-IR neurons was similar in HSCR without OS as compared to ARM patients. These observations suggest that the reduction in nNOS neurons is specific to HSCR patients with OS, as continence disorders observed in ARM patients are probably related to distinct underlying mechanisms, such as defects of the pelvic muscular complex, including the anal sphincter. Concerning the changes in nNOS neurons in HSCR, the vast majority of studies have identified a reduced number of nNOS-IR neurons or a reduced nitrergic innervation in the aganglionic segments^11,12^. Only recently, Cheng et al. observed changes in nNOS neurons in the proximal resection margin of HSCR patients. These changes were characterised by an increased proportion of nNOS myenteric neurons (nNOS +/Tuj + neurons) as compared to “controls”^13^. When expressed in proportion of neurons (normalised to the total number of neurons), we also observed an increased proportion of nNOS neurons in HSCR patients as compared to ARM patients. Similarly an increased proportion of nNOS myenteric neurons was also described in the distal bowel of mice models of HSCR^14,15^. However, the increased proportion of nNOS myenteric neurons reflects probably the fact that the number of nNOS neurons is less reduced than the total number of neurons in HSCR patients. Furthermore, in our study, the proportion of nNOS neurons was significantly reduced in HSCR patients with OS as compared to HSCR patients without OS. Cheng et al. showed that the increased proportion of nNOS neurons in HSCR patients was retrospectively associated with non-stratified postoperative complications, including indifferently constipation, HAEC and faecal incontinence^13^. These differences between our studies result from the fact that they did not stratify patients according to the nature of postoperative complications (OS, HAEC or diarrhoea, that are discriminated by the proportion of nNOS neurons in our study). Moreover, they included a limited number of “control” patients with a large individual variability in terms of age and underlying diseases (2 chronic constipation, 1 ARM, 1 sigmoid stricture after necrotizing enterocolitis). Interestingly, besides showing a reduced total number of neurons and nNOS-IR neurons, HSCR patients with OS also had a reduced TLR2 and TLR4 mRNA expression as compared to ARM patients. In addition, we observed a strong correlation between TLR2/4 expression and the number of nNOS myenteric neurons. Altogether, it is tempting to speculate that the reduced TLR2/4 expression could have contributed to the alteration of ENS phenotype, as TLR2−/− and TLR4−/− mice have respectively a reduction in the total number of neurons and nNOS-IR neurons^10,16^.

Another major finding of our study is that HSCR patients with complications present alterations of functional parameters associated with a remodelling of the ENS at the time of curative surgery, in particular with a reduced expression of nNOS neurons. Although the NO-mediated response was previously reported to be significantly reduced or absent in the aganglionic bowel as compared to the normoganglionic one^17^, no change of this response has been reported in the normoganglionic bowel up until now. In particular, in our study, the NO-mediated neuromuscular response was different between ganglionic areas of complicated and uncomplicated HSCR, and between HSCR and ARM patients. In this context, alterations of the nitrergic neuromuscular transmission in the longitudinal muscle may contribute in part to persistent motility disorders in HSCR patients, as NO is a major neuromediator involved in the peristaltic activity of the gut^18–22^. Therefore, approaches aimed at restoring the nitrergic phenotype could be of major preventive and therapeutic interest for HSCR patients. Among targets of interest, enema of butyrate has been shown to increase the proportion of nitrergic neurons (and also of cholinergic ones), leading altogether to an enhanced colonic transit time^23,24^. However, increasing solely the nitrergic population might be taken with caution as, in a recent study in *TashT*^Tg/Tg^ mice, the authors suggested that the increased proportion of nitrergic neurons could play an important role in the colonic dysmotility^25^.

A third major finding of this study was that the patients who developed postoperative HAEC in the follow-up had a significant increased transcellular and paracellular permeability at the time of curative surgery as compared to other HSCR subgroups, and also to ARM patients. This suggests that altered intestinal barrier functions are present early in life and could facilitate the development of HAEC later in life. We did not identify molecular targets, in particular TJ that could be potentially involved in observed changes in barrier functions. Indeed, we did not observe changes in major TJ proteins expression. However, we can not exclude that an altered expression of other non-studied TJ proteins or even a TJ cellular redistribution^26^ could occur and directly contribute to the observed changes in permeability. Interestingly, a recent study showed that in GFRα-1 hypomorphic mice, HAEC was preceded and associated with diffuse epithelial hyperplasia, as well as dysplasia, and accompanied by degeneration of surface colonocytes and goblet cell dysplasia^27^. However, the authors did not evaluate the functional impact of these changes upon paracellular or transcellular permeability. Conversely, whether similar changes occur in HSCR is unknown and deserves future studies. In our study, surprisingly, increased permeability in HSCR patients that develop HAEC was not associated with increased cytokine expression. In contrast, we found a reduction in TNFα mRNA and in TLR2/TLR4 mRNA expression in this subgroup of patients with an increased permeability as compared to ARM. Previous studies have also reported, early in the postnatal period, an absence of major changes in cytokines expression in mice models predisposed to develop HAEC^27^. Therefore, as in our study HAEC developed months after surgery, we might have been to early to detect any changes in cytokine expression. Altogether our results suggest that reinforcing intestinal barrier functions in this subpopulation of HSCR patients would therefore be of great putative interest to prevent HAEC later in life.

Finally, our study has also revealed glioplastic changes characterised by an up-regulation of GFAP N-term in the ganglionic zone of HSCR patients as compared to ARM. In the ENS, several isoforms of GFAP are expressed and changes in their expression have been reported in inflammatory bowel disease^28^, Chagas disease^29^, intestinal atresia^30^, slow transit constipation^31^ and chronic intestinal pseudo-obstruction^32^. In our case, changes in expression of GFAP N-term isoform could reflect an altered glial maturation, as GFAP expression increases during the early neonatal period^33^. Interestingly, Musser et al^14^ showed, in a Sox10+/− mouse with HSCR, an increase in EGC population, inversely correlated with the severity of the neuronal cell loss. It remains to be determined whether such changes reflect an adaptive response of EGC to compensate for the neuronal cell loss^34^.

We are perfectly conscious that one limitation of our study, and also of other previously published studies, is the use of ARM as a comparative group. Nevertheless, access to normal colonic tissues in neonates is extremely challenging. In ARM, enterocolitis is not described in the postoperative evolution and continence disorders are mainly related to defects of the pelvic muscular complex. It remains unestablished whether ARM patients present abnormalities of the ENS because the ENS has never been studied specifically in the segment of sigmoid colon we used^35^. In addition, in our ARM patients, this sigmoid colon presented morphological and phenotypic changes in the ENS that have been previously reported in healthy newborn animals^36^. In particular, we found an age-dependent increase in neuronal cell body size and in the number of nNOS neurons in ARM, but not in HSCR patients (data not shown).

In summary, we found a significant reduction in the number of nNOS enteric neurons in the ganglionic segments of HSCR patients who presented OS in the postoperative evolution, and a significant increase in IEB permeability at the time of surgery in the ganglionic segments of HSCR patients who presented HAEC in the postoperative evolution. In this context, if confirmed in larger studies, our data could set the basis for developing novel personalised therapeutic approaches to modulate the ENS phenotype and enhance the intestinal barrier functions in order to prevent or treat not only postoperative complications in HSCR.

## Methods

### Patient inclusion

Eighteen HSCR patients, with a diagnosis of short-segment HSCR (limited to rectum or sigmoid colon) born after 37 weeks of gestation and aged less than one year at the time of curative surgery, were included from eight paediatric surgery departments (10 paediatric surgeons) from July 2014 to May 2016. Curative surgery was a transanal pull-through procedure with an anastomosis in the ‘healthy’ ganglionated zone, defined according to histopathological consensus criteria after evaluation of the entire circumference of the proximal margin: ganglion cells in submucosal and myenteric plexi on the entire circumference of the proximal part of the resected bowel (> 7/8th circumference), with normoganglionic myenteric plexi and without hypertrophied nerve fibres^37^. A double histopathological reading by two Pathologists with an expertise in HSCR provided the confirmation that the anastomosis of the transanal pull-through procedure was performed in the normoganglionated zone. Non-inclusion criteria were: preoperative enterocolitis, anastomosis in the transition zone, another intestinal malformation associated with gastrointestinal (GI) dysmotility and severe associated malformations (trisomy 21) or associated diseases (hypothyroidism, cystic fibrosis) that may impact on intestinal motility.

Sixteen patients with a diagnosis of ARM (high ARM in males with recto-bulbar or recto-prostatic fistula; “intermediate” ARM in females with recto-vestibular fistula) requiring a colostomy creation or closure before one year of age and born after 37 weeks of gestation were included and considered in our study as a comparison group. The colostomy was always performed at the level of the sigmoid colon.

### Prospective clinical follow-up

HSCR patients were prospectively followed at 3, 6, 9, 12, 18, 24 and 36 months after surgery by the paediatric surgeon who performed the surgery using a standardized interview recorded in a Case Report Form and adapted to the age of the patient. Functional intestinal OS were recorded by parents and during medical examination. The age of onset and the treatment received (regardless of its efficiency) were also recorded. Stool consistency was assessed after dietary diversification at onset of symptoms using the Amsterdam stool form scale for not-toilet-trained infants (A = watery, B = soft, C = formed, D = hard)^6^. Postoperative functional intestinal OS were defined as the presence or the association of the following symptoms: constipation, straining with defecation, stool retention in the colon, abdominal distension^4^. Constipation was defined by hard stool consistency as a mandatory criterion (type D) with two or fewer defecations per week and/or large faecal mass in the colon at abdominal palpation or rectal examination^38^. Stool retention in the colon was defined as the storage of stools in the colon with a maintained defecation every day and with faecal masses at abdominal palpation. The functional character of OS was assessed after exclusion of postoperative surgical technical complications: mechanical obstruction (anastomotic stricture, twist), persistent aganglionosis or transition zone at the site of the anastomosis^7^. Episodes of HAEC were diagnosed according to the HAEC score (HAEC score ≥ 4) and severity was graded from 1 to 3 according to published guidelines^3,8^. Diarrhoea was defined by more than 4 liquid stools per day (type A) after dietary diversification without OS^39,40^.

ARM patients were also followed prospectively and enterocolitis was noted following the same diagnostic criteria, but OS were not collected because of continence disorders inherent to this developmental malformation related to defects in the pelvic muscular complex (including the anal sphincter).

### Sigmoid colon tissue collection and preparation

Specimens of resected bowel were collected at the time of surgery from the ganglionated zone in HSCR and from the proximal part of the stoma (sigmoid colon) in ARM. They were placed immediately in sterile Hank’s balanced salt solution (HBSS) kept at 4 °C on ice. Mucosa and submucosa were separated from the muscular tissue by microdissection. Samples were placed immediately in liquid nitrogen, stored at − 80 °C or maintained at 4 °C on ice in sterile HBSS and transported to the laboratory in less than 5 h.

Only one paediatric surgeon collected and conditioned all the specimens (following standardised procedures developed before the beginning of the study), and then performed all the following experimental studies that were analysed prior to the collection of the prospective clinical data.

### Immunofluorescence staining

Samples of muscular preparations were pinned and stretched in a dissection dish, then fixed in 0.1 M phosphate buffered saline (PBS) containing 4% paraformaldehyde for 3 h at room temperature, as previously described^36^. After three PBS washes, the circular muscle was removed under microscope to expose the myenteric plexus (MP). The longitudinal muscle/myenteric plexus (LMMP) preparation was permeabilised for 1 h in PBS/NaN_3_ (0.1%) containing 0.5% TritonX-100 and 4% horse serum. LMMPs were incubated with primary antibodies for 18 h at 4 °C (Table 2). The tissues were washed with PBS and incubated for 2 h with donkey anti-mouse IgG FluoProbes488 (1:100; INTERCHIM, Montluçon, France) or anti-rabbit IgG Cy3 (carboxymethylindocyanine) (1:500; JACKSON IMMUNORESEARCH, West Grove, PA, USA). After PBS washes, specimens were viewed under an Olympus IX 50 fluorescence microscope fitted with suitable filter cubes. Images were acquired with a digital camera (model DP 71; OLYMPUS, Rungis, France) coupled to the microscope, using Cell B software (Soft Imaging System; Olympus) and analysed with Image J software.

**Table 2:** Primary antibodies.

Antibody	Host	Source or reference	Dilution	Method
HuC/D	Mouse	INVITROGEN, USA (A-21271)	1:200	Immunofluorescence
nNOS	Rabbit	ALEXIS BIOCHEMICALS, USA (ALX-210-501)	1:2000	Immunofluorescence
Calretinin	Rabbit	INVITROGEN, USA (18-0211)	1:500	Immunofluorescence
ZO-1	Rabbit	THERMOFISHER SCIENTIFIC, USA (40-2200)	1:200	Western Blot
Occludin	Rabbit	ABCAM, USA (ab167161)	1:50,000	Western Blot
Claudin-1	Rabbit	ABCAM, USA (ab180158)	1:200	Western Blot
JAMA	Rabbit	BETHYL LABORATORIES, USA (A 302891)	1:1000	Western Blot
Cingulin	Rabbit	SANTA CRUZ BIOTECHNOLOGY, USA (sc-66831)	1:500	Western Blot
panGFAP	Rabbit	DAKO, France (Z0334)	1:2000	Western Blot
GFAP N-term	Mouse	SCBT, USA (F-2)	1:500	Western Blot
β-Actine	Mouse	SIGMA	1:10,000	Western Blot

*HuC/D* human neuronal protein, *nNOS* neuronal nitric oxide synthase, *GFAP* glial fibrillary acidic protein.

Five randomly selected fields of LMMP per patient were examined. ENS area occupied by tissue surface unit was defined by measuring the surface of calretinin-immunoreactive (IR) structures (interganglionic strands and ganglia) and expressed as a percentage of the field area observed with the objective × 10 (893.8 × 673 µm = 601,527.4 µm^2^). Neuronal cytoplasmic area was defined by measuring the surface occupied by the cytoplasm of enteric neurons in myenteric plexuses using HuC/D staining. The number of HuC/D-IR, nNOS-IR and calretinin-IR myenteric neurons per ganglion were counted (at least 500 neurons per preparation).

### Ex vivo measurements of neuromuscular transmission

Strips of fresh circular and longitudinal muscles were placed in organ chambers (RADNOTI LLC, Monrovia, CA USA) containing 15 mL of Krebs solution at 37 °C continuously bubbled with 95% O_2_ and 5% CO_2_. Their contractile responses were recorded using isometric force transducers (No. TRI02PAD, PANLAB, Barcelona, Spain) coupled to a computer equipped with the PowerLab 8/30 System and the Labchart data analysis software (AD INSTRUMENTS, Spechbach, Germany), as previously described^24^.

Strips were stretched with a preload of 1 g maintained during an equilibration period of 90 min and subjected to EFS using a STG4008MCS electrical stimulator (MULTI CHANNEL SYSTEMS, Reutlingen, Germany) to activate enteric neurons (train duration: 10 s; pulse frequency: 20 Hz; pulse duration: 400 µs; pulse amplitude: 11 V). The contractile activity was evaluated by measuring the EFS-induced AUC during the EFS period (10 s). Basal EFS-induced AUC was first assessed. In order to characterise the nitric oxide (NO)-dependent EFS-induced relaxation, the same protocol was used with NO synthase inhibitor l-NAME (50 µM, SIGMA-ALDRICH, St Louis, MO, USA) and atropine (1 µM, SIGMA-ALDRICH), an antagonist of cholinergic muscarinic receptors, to characterise the cholinergic-dependent EFS-induced contraction. Response to l-NAME and atropine were defined by the variation of EFS-induced AUC (expressed in percentage) with l-Name and with atropine (each patient acted as their own control). The variation of EFS-induced AUC with l-Name was the difference between AUC in presence of l-Name and AUC in basal condition, normalized to AUC in basal condition. The variation of EFS-induced AUC with l-Name  + atropine was the difference between AUC in presence of l-Name  + atropine and AUC in presence of l-Name, normalized to AUC in presence of l-Name.

### Acetylcholine assay

Muscular tissues were lysed in RIPA lysis buffer (MILLIPORE, Burlington, MA, USA) with sodium orthovanadate (SIGMA-ALDRICH), phosphatase inhibitors (Phosphatase inhibitor cocktail 3; ROCHE, Boulogne-Billancourt, France) and protease inhibitors (Complete; ROCHE) using a Precellys 24 tissue homogeniser (BERTIN TECHNOLOGIES, Aix-en-Provence, France), followed by sonication with a Vibracell 75186 (SONICS, Newton, CT, USA). Total protein levels were quantified using a bicinchoninic acid protein assay kit (THERMOFISHER, Waltham, MA, USA). Acetylcholine concentration was determined in tissue homogenates containing equal amounts of proteins (75 µg) (Amplex red acetylcholine/acetylcholinesterase assay kit; THERMOFISHER).

### Ex vivo para- and transcellular permeability

Samples of mucosa were mounted in Ussing chambers (PHYSIOLOGICAL INSTRUMENTS, San Diego, CA, USA) exposing a surface of 0.0314 cm^2^, as previously described^24^. Tissues were bathed on each side with 2 mL of F12 Dulbecco’s Modified Eagle medium (THERMOFISHER) containing 0.1% (v/v) fetal bovine serum. The medium was oxygenated continuously and maintained at 37 °C (95% O_2_/5% CO_2_). After a 30 min equilibration period, 275  µL of apical medium was replaced with 200 µL of sulfonic acid (SA) solution (400 Da, final concentration 0.1 mg/mL) (THERMOFISHER) and 75  µL of horseradish peroxidase (HRP) solution (final concentration 0.375 mg/mL) (SIGMA-ALDRICH) to assess paracellular and transcellular permeabilities. The SA fluorescence level of the basolateral chamber was measured every 30 min over 3 h from the luminal surface using a fluorimeter (Varioskan; THERMOFISHER). The enzymatic activity of HRP was also measured every hour over 3 h by colorimetric enzymatic activity assay using 3,3′-5,5′-tetramethylbenzidine (BD BIOSCIENCE, Le Pont-de-Claix, France). The slope of fluorescence intensity and HRP activity over time was calculated to determine paracellular and transcellular permeabilities, respectively.

### Western Blot

Mucosa with submucosa and muscular tissues were lysed and quantified as previously described^9^. Equal amounts of lysate (10 µL containing 10 µg of proteins) were separated using the INVITROGEN NuPage Novex 4–12% Bis–Tris MidiGels together with Bis–Tris running buffer before electrophoretic transfer to a nitrocellulose membrane with iBlot Dry Blotting System (THERMOFISHER). Membranes were placed in a 10% acetic acid bath for 10 min, then washed with Tris-buffered saline (TBS) (150 mM NaCl, 15 mM Tris, 4.6 mM Tris Base, pH 7.4) and blocked overnight at 4 °C in TBS with 5% non-fat dry milk. Membranes were incubated overnight at 4 °C with primary antibodies (Table 2). Bound antibodies were detected with anti-rabbit and anti-mouse HRP-conjugated antibodies (1:5000; THERMOFISHER) and visualised by enhanced chemiluminescent detection (ECLPRIME, Amersham, France). The relevant immunoreactive bands were quantified with laser-scanning densitometry and analysed with ImageLab Software (BIO-RAD, Marnes-la-Coquette, France). Values were normalised to the amount of beta-actin. Quantitative comparisons were performed on the same blot and only one blot was performed for each antibody to avoid comparison between different blots (Supplementary material).

### Quantitative polymerase chain reaction analysis

Total RNA was extracted from the samples of mucosa/submucosa crushed in 600 µL of RA1 and 1/100 β-mercaptoethanol with Precellys 24 tissue homogeniser (BERTIN TECHNOLOGIES) using nucleospin RNA II (MACHEREY-NAGEL, Hoerdt, France), as previously described^33^. Potential genomic DNA contamination was removed by treatment with Turbo DNase (AMBION INC, Austin, TX, USA) and RNA was quantified using a Nanodrop 200° ND-1000 UV–vis spectrophotometer (NANODROP TECHNOLOGIES, Wilmington, NC, USA). Samples were processed to obtain 1 µg RNA in a final volume of 15 µL. The reverse transcriptase reaction was realised from 1 µg total RNA incubated at 72 °C for 3 min using the Super Script III Reverse Transcriptase System kit (THERMOFISHER) in the Thermal Cycler 2720 thermocycler (APPLIED BIOSYSTEM, Foster City, CA, USA) (25 °C for 5 min, 50 °C for 55 min, 70 °C for 15 min). The cDNA was diluted to a final concentration of 0.8 ng equivalent RNA/µL. The sense and anti-sense oligonucleotide primers used in this study are shown in Table 3.

**Table 3:** Primers for qPCR analysis.

Primer	Gene	Sequence (5′–3′)
h-COX1	Cyclooxygénase 1	Up-CACCCATGGGAACCAAAG
		Lp-TGGGGGTCAGGTATGAACTT
h-COX2	Cyclooygénase 2	TGGGAAGCCTTCTCTAACCTC
		TCAGGAAGCTGCTTTTTACCTT
h-HPGDS	Hematopoietic Prostaglandin D synthase	GAGAATGGCTTATTGGTAACTCTGT
		AAAGACCAAAAGTGTGGTACTGC
h-LPGDS	Lipocalin prostaglandin D synthase	AGAAGAAGGCGGCGTTGTCC
		CCACCACTGACACGGAGTAGG
h-MPGES1	Microsomal prostaglandin E sykthase-1	CGCTGCTGGTCATCAAGA
		CGTGTCTCAGGGCATCCT
h-TLR2	Toll-like receptor 2	CGTTCTCTCAGGTGACTGCTC
		CCTTTGGATCCTGCTTGC
h-TLR4	Toll like receptor 4	CCATGGCCTTCCTCTCCT
		TCAGCTCCATGCATTGATAAGT
h-TLR9	Toll like receptor 9	TGTGAAGCATCCTTCCCTGT
		AGAGACAGCGGGTGCAG
h-IL1beta	Interleukin beta 1	GAGCAACAAGTGGTGTTCTCC
		TTGGGATCTACACTCTCCAGC
h-IL8	Interleukin beta 8	CTGGCCGTGGCTCTCTTGG
		ATTTCTGTGTTGGCGCAGTGTG
h-IL10	Interleukin beta 10	TGAAAACAAGAGCAAGGCCG
		GCCACCCTGATGTCTCAGTT
h-TNFalpha	Tumor necrosis factor alpha	CCCGAGTGACAAGCCTGTAG
		TGAGGTACAGGCCCTCTGAT
h-IFNgamma	Interferon gamma	CCAGAGCATCCAAAAGAGTGTGGAG
		GCTGGCGACAGTTCAGCCATCA

Amplifications were performed in duplicate using StepOne Plus (THERMOFISHER) detection system with Fast SYBR Green (THERMOFISHER) master mix. A standard curve was generated with serial dilutions of control cDNA by plotting the relative amounts of these dilutions against the corresponding Ct (cycle threshold) values. The level of expression of each gene was calculated from these standard curves using StepOneplus software. Expression of S6 ribosomal protein was used as a reference gene. For each sample, the ratio between the relative amount of each specific transcript and S6 was calculated to compensate variations in mRNA. Samples were tested in duplicate and the mean values were used for quantification using the 2^-ddCt method.

### Statistical analysis

Figures and statistical analysis were performed using Prism Software (GRAPHPAD). Data were expressed in medians (extremes). Non-parametric tests were used. Comparison and correlation of data for HSCR and ARM were performed using Mann-and-Whitney U test and Spearman correlation. A two-way repeated measure ANOVA was used to compare the three HSCR subgroups and the ARM comparison group. Differences were considered significant at *p* < 0.05.

### Study approval

Human samples were collected after obtaining a written informed consent from either the parents or the legal guardians prior to inclusion in the study. This study received IRB approval by Nantes Ethical Committee under protocol number 12-16-2014. The database is in compliance with the requirements of the French data protection authority (CNIL, 08/07/2015-no. 915,187) and registered by the Research Ministry under protocol 03/05/2015-no. 15.071bis. The EnteHirsch biobank was approved by the Clinical Ethics Committee (CPP Ouest IV of Nantes, 11/09/2014-no. DC-2011-1399). All methods were carried out in accordance with relevant guidelines and regulations.

## Supplementary information

Supplementary Figure S1.
