# Supplementary material for: Analysis of enteric nervous system and intestinal epithelial barrier to predict complications in Hirschsprung’s disease
Source: Sci Rep. 2020 Dec 10;10:21725. doi: 10.1038/s41598-020-78340-z (PMC7729910; doi:10.1038/s41598-020-78340-z)
Supplement: Supplementary file 1 — Supplementary Figure S1. [file 41598_2020_78340_MOESM1_ESM.pdf]

Supplemental information

Analysis of Enteric Nervous System and Intestinal Epithelial Barrier to Predict Complications in Hirschsprung's Disease

Anne Dariel[1,2,3 *], Lucie Grynberg[1], Marie Auger[1], Chloé Lefèvre[1], Tony Durand[1], Philippe Aubert[1], Catherine Le Berre-Scoul[1], Aurélien Venara[1], Etienne Suply[1], Marc-David Leclair[3], Philine de Vries[4], Guillaume Levard[5], Benoit Parmentier[5], Guillaume Podevin[6], Françoise Schmitt[6], Véronique Couvrat[7], Sabine Irtan[8], Erik Hervieux[8], Thierry Villemagne[9], Hubert Lardy[9], Carmen Capito[10], Cécile Muller[10], Sabine Sarnacki[10], Jean-François Mosnier[11], Louise Galmiche[1,12], Hélène Boudin[1], Pascal Derkinderen[1], Charlène Brochard[1], Michel Neunlist[1]

[1] University of Nantes and University Hospital of Nantes, INSERM UMR1235, TENS, The Enteric Nervous System in Gut and Brain Diseases, IMAD, France
[2] Paediatric surgery department, La Timone-Enfants Hospital, Assistance Publique des Hôpitaux de Marseille, France
[3] Paediatric surgery department, University Hospital of Nantes, France
[4] Paediatric surgery department, University Hospital of Brest, France
[5] Paediatric surgery department, University Hospital of Poitiers, France
[6] Paediatric surgery department, University Hospital of Angers, France
[7] Paediatric surgery department, Hospital of Le Mans, France
[8] Paediatric surgery department, Armand Trousseau Hospital, Paris, France
[9] Paediatric surgery department, University Hospital of Tours, France
[10] Paediatric surgery department, Necker Enfants Malades Hospital, Paris, France
[11] Pathology department, University Hospital of Nantes, France
[12] Pathology department, Necker Enfants Malades Hospital, Paris, France

S1. Full length blots of glial fibrillary acidic protein (GFAP) expression in the muscular layer of sigmoid colon in HSCR and ARM patients. Three blots from the same membrane using one antibody recognizing all GFAP isoforms and trunked forms of the protein (panGFAP), one specific antibody recognizing intact GFAP isoforms (GFAP N-term) and one antibodie recognizing β-Actin. The red boxes denote the regions used in the corresponding main figure 3. (OS : obstructive symptoms ; HAEC/D : Hirschsprung-associated enterocolitis/diarrhea ; w/oC : without complications ; ARM : anorectal malformation)

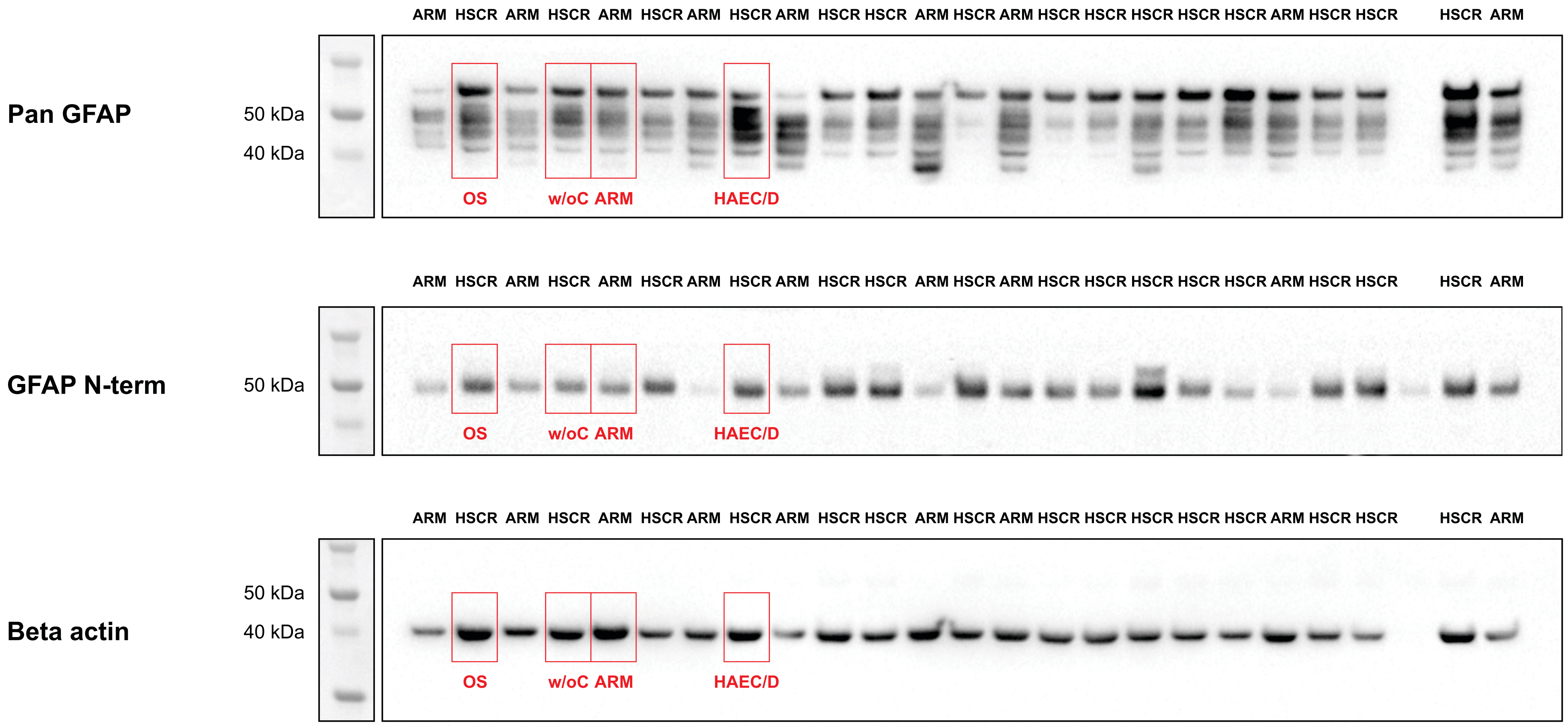

Pan GFAP
GFAP N-term
Beta actin
50 kDa
40 kDa
ARM HSCR ARM HSCR ARM HSCR ARM HSCR ARM HSCR HSCR ARM HSCR ARM HSCR HSCR HSCR HSCR HSCR ARM HSCR HSCR HSCR ARM
OS
w/oC ARM
HAEC/D
